# Equidistance between Primary Sensory-Motor Landmarks Shapes Functional Specialization in the Human Brain

**DOI:** 10.1523/JNEUROSCI.0352-26.2026

**Published:** 2026-09-24

**Authors:** Lidón Marin-Marin, Xiuyi Wang, Jonathan Smallwood, Daniel S. Margulies, Elizabeth Jefferies

**Affiliations:** ^1^Department of Psychology, University of York, York YO10 5NA, United Kingdom; ^2^York Neuroimaging Centre, Innovation Way, York YO10 5NY, United Kingdom; ^3^Departament de Psicologia Evolutiva, Educativa, Social i Metodologia, Universitat Jaume I, Castelló de la Plana 12006, Spain; ^4^State Key Laboratory of Cognitive Science and Mental Health, Institute of Psychology, Chinese Academy of Sciences, Beijing 100101, China; ^5^Institute of Psychology, Chinese Academy of Sciences, Beijing 100101, China; ^6^Department of Psychology, University of Chinese Academy of Sciences, Beijing 100049, China; ^7^Department of Psychology, Queen’s University, Kingston, Ontario K7L 3N6, Canada; ^8^Oxford Centre for Integrative Neuroimaging (OxCIN), FMRIB, Nuffield Department of Clinical Neurosciences, University of Oxford, Oxford OX3 9DU, United Kingdom; ^9^Centre National de la Recherche Scientifique (CNRS), Oxford UAR 3129, United Kingdom

**Keywords:** brain activation, connectivity gradient, equidistance, functional specialization, geodesic distance, primary landmarks

## Abstract

The human cortex is organized around primary sensory-motor landmarks, but the way these distances shape large-scale functional organization remains unclear. Using geodesic measures in males and females, we show that global minimum distance to sensory-motor landmarks aligns with the principal gradient of intrinsic connectivity, reflecting the shift from unimodal to heteromodal cortex, while equidistance between landmarks captures complementary organizational patterns. Heteromodal networks, including default mode, control, and limbic systems, occupy cortical regions that are both distant from primary systems and relatively balanced between them. Subsystems within these networks exhibit distinctive spatial embeddings: core default mode regions sit equidistant between visual and somatomotor cortex, frontotemporal default mode regions favor auditory cortex but remain relatively balanced, and medial temporal regions are biased toward visual cortex. Control network subsystems also vary: the control-DMN subsystem occupies the most distant and equidistant positions, core control is closer to motor regions, and medial control is closer to visual cortex, reflecting their different functional specializations. At the parcel level, greater equidistance predicts stronger task-evoked activation, particularly in heteromodal border zones. These findings highlight two complementary spatial principles—proximity and balance—that structure the cortex, shaping both the location and functional profile of heteromodal networks.

## Significance Statement

Distance from unimodal cortex has been proposed as a structural constraint on functional organization. However, it remains unclear how relative position with respect to multiple sensory-motor landmarks contributes to functional specialization. Here, we show that both absolute distance from primary landmarks and their relative balance (equidistance) shape the organization of heteromodal networks. Equidistance between specific sensory-motor landmarks differentiates heteromodal subsystems and predicts task-evoked activation during story comprehension and mathematical reasoning. These results demonstrate that cortical geometry is linked to cognitive function and highlight two complementary spatial principles—proximity and balance—that structure the cortex and shape the functional profile of heteromodal networks.

## Introduction

Brain function reflects the anatomical organization of the cortex. Classical models of cortical hierarchies (Mesulam, 1998) and gradient-based frameworks (Margulies et al., 2016) emphasize the role of sensory-motor landmarks in shaping large-scale organization. The principal gradient of functional connectivity reflects an axis from sensory-motor to transmodal association regions, capturing the sequence of large-scale networks from default mode (DMN), through control and attention networks to primary regions (Yeo et al., 2011). This principal gradient correlates with geodesic distance from primary landmarks—the central sulcus, calcarine sulcus, and temporal transverse sulcus—highlighting distance from unimodal cortex as a structural determinant of functional organization.

Distance, however, can be conceptualized in different ways. Global minimum distance indexes proximity to the nearest sensory-motor landmark, whereas equidistance reflects whether a parcel lies at similar distances from multiple landmarks, potentially identifying integrative zones between unimodal systems. These metrics capture distinct spatial principles—absolute proximity versus relational balance—that may have different consequences for brain function.

The positioning of heteromodal networks illustrates this distinction. DMN and limbic regions are thought to support memory-guided and multimodal cognition via their separation from sensory-motor cortex, fostering abstract and decoupled representational states (Smallwood et al., 2021; Wei et al., 2024). At the same time, theories of semantic integration propose that regions such as the anterior temporal lobe act as graded hubs, combining distance from unimodal systems with equidistance between visual and auditory inputs for balanced conceptual abstraction (Lambon-Ralph et al., 2017). Likewise, the multiple-demand network (MDN), recruited across diverse tasks (Duncan, 2010), flexibly adapts to different input types (Shashidhara et al., 2024), suggesting that equidistance to multiple systems might facilitate balanced integration. Thus, distance may support abstraction, while equidistance may support multimodal convergence. Crucially, these spatial principles are not interchangeable: a region may be equidistant from two landmarks yet close to both, or far from both.

Empirical evidence underscores the functional impact of spatial embedding for the visual modality (Klatzmann et al., 2025). Similarly, Wang et al. (2023) showed that auditory parcels closer to sensory-motor landmarks activate more during story listening, whereas DMN parcels farther from landmarks also show stronger activation, suggesting that proximity supports efficient stimulus processing while distance facilitates semantic processing. Similarly, Benn et al. (2025) found that reading performance is predicted by shorter distances between visual cortex and transmodal regions. The consequences of equidistance, however, remain poorly understood. Because equidistance indexes balance rather than hierarchy, it may capture localized patterns of integration rather than large-scale gradients. Related work supports this view: parcels at the boundaries of MDN encode more information about stimulus categories than core MDN regions, which track task structure (Shashidhara et al., 2024). This suggests that intermediate spatial positioning may enable flexible access to both task-general and stimulus-specific information.

Equidistance may also vary within distributed networks. Although DMN and MDN are broadly distant from sensory-motor inputs, their subsystems occupy distinct cortical locations: DMN can be fractionated into core, medial temporal (MT), and frontotemporal (FT) subsystems (Yeo et al., 2011), each linked to specialized functions such as embodied simulation, scene construction, and social reasoning (Andrews-Hanna et al., 2010; Zhang et al., 2022; Gonzalez Alam et al., 2024). Likewise, the control network divides into subsystems with distinct coupling to DMN or dorsal attention networks (Dixon et al., 2018). These divisions imply that subsystems may exploit different combinations of distance and equidistance to optimize their roles—a subsystem biased toward visual cortex may support imagery, while another balanced across landmarks may support integrative reasoning.

In this study, we examine the relevance of two spatial metrics, global minimum distance and equidistance, to large-scale functional organization, including the hierarchy from unimodal to transmodal processing captured by the principal gradient and the organization of DMN and control systems into specialized subnetworks. We further test whether spatial embedding predicts functional activation during cognitive tasks. By linking proximity and relative balance to both intrinsic gradients and task responses, we aim to clarify how cortical geometry supports abstraction, integration, and cognitive flexibility.

## Materials and Methods

### Experimental design

#### Dataset and participants

The main dataset was obtained from the Human Connectome Project (HCP; Glasser et al., 2013) and consisted of 1,039 healthy volunteers (483 males, 556 females), aged 22–37 years (mean = 28.74, SD = 3.69). Detailed descriptions of MRI acquisition, preprocessing, analysis, and parcellation of the HCP dataset have been provided previously (Binder et al., 2011; Barch et al., 2013; Glasser et al., 2013, 2016). All participants provided their informed consent, and the study was approved by the Institutional Review Board of Washington University at St. Louis.

#### Minimum geodesic distance to landmarks

Following the approach used in a previous investigation (Wang et al., 2023), we based our analyses on the HCP multimodal parcellation map 1.0 (Glasser et al., 2016). This parcellation consists of 180 brain areas per hemisphere, obtained by using a semiautomated neuroanatomical approach on magnetic resonance images of 210 healthy young adults from the HCP (for details, see Glasser et al., 2016; Wang et al., 2023).

We obtained the minimum geodesic distance between each parcel of the HCP multimodal parcellation and key landmarks of primary visual, auditory, and somatomotor cortex from a previous investigation (Wang et al., 2023). The landmarks used were the following: (1) central sulcus, corresponding to the primary somatomotor cortex; (2) temporal transverse sulcus, for the primary auditory cortex; and (3) calcarine sulcus, for the primary visual cortex.

The primary landmarks were defined using an automated procedure in FreeSurfer, by which surface mesh representations of the cortex from individual subjects' T1 images are reconstructed. Specifically, the cortical surface mesh of each participant was inflated into a sphere and registered to a common spherical coordinate system that aligns cortical folding patterns across subjects, obtaining a nonlinear mapping between the native T1 space and fsaverage surface space (Dale et al., 1999; Fischl et al., 1999). Then, corresponding surface parcellations of 74 sulci and gyri were generated for each subject, based on probabilistic and geometric information derived from the cortical model of the subject (Fischl et al., 2004; Desikan et al., 2006; Destrieux et al., 2010). After that, each individual surface was resampled from fsaverage space into fs_LR ∼32k space, obtaining all vertices that belong to the three landmarks of interest. More information can be found in a previous publication (Wang et al., 2023). Then, for each participant, the Connectome Workbench software was used to calculate the geodesic distance between all pairs of vertices in the brain along the “midthickness” of the cortical surface (halfway between the pial and white matter), using an algorithm measuring the shortest path between two vertices on a triangular surface mesh. After surface-based registration and removal of vertices representing the medial wall to ensure that the shortest paths would only pass through the cortex, distance values independent of mesh density were obtained. Finally, minimum geodesic distance between vertices and each landmark was found after calculating the distance between each vertex *i* outside the landmark and each vertex *j* within the landmark and then choosing the pair of vertices that were closer. To calculate the minimum geodesic distance between each parcel in the HCP multimodal parcellation (Glasser et al., 2016) and each landmark, the average minimum distance across all vertices in parcel *k* to the vertices within the landmark was calculated. A detailed description of this process can be found in Wang et al. (2023).

In order to explore if distance measures were stable across participants, we quantified the across-subject stability of the parcelwise geodesic distance maps. Specifically, for each participant and each landmark, we computed the spatial correlation (Pearson) between that participant's parcelwise geodesic distance map (raw values, not normalized) and the leave-one-out group-average distance map derived from all other participants. This showed high consistency across individuals (central sulcus: mean correlation = 0.994 ± 0.004, range = 0.935–0.998; calcarine sulcus: mean correlation = 0.995 ± 0.002, range = 0.978–0.998; temporal transverse sulcus: mean correlation = 0.994 ± 0.003, range = 0.974–0.998). This suggests that the parcelwise geodesic distance measures were stable across participants, while still preserving individual anatomical variability (see Fig. S4 for a visual representation of the variation in geodesic distance across participants of three parcels relevant for our results). Finally, we explored the reliability of the between-subject variability in raw geodesic distance using the intraclass correlation coefficient (Shrout and Fleiss, 1979) and based our approach on the high levels of anatomical symmetry between hemispheres (Kuo and Massoud, 2022). This resulted in fair to good reliability for parcelwise distance and excellent reliability for average distance within hemisphere (Cicchetti, 1994; Koo and Li, 2016; see Fig. S4’s text for details).

#### Global minimum distance and equidistance

After identifying the minimum geodesic distances between each parcel and the three primary landmarks (Fig. 1*A*), we then selected the lowest value among the three for each parcel—representing the lowest distance between parcel *k* and any landmark. We used it to define the global minimum distance to any primary region for parcel *k* (Fig. 1*B*). Values of global minimum distance ranged from 0 to 203.3. Global minimum distance values were normalized within each participant to a range between 0 and 1 using the min-max scaling method (MinMaxScaler) of the scikit-learn library and reversed (resulting in 0 = most distant from any landmark, 1 = closest) for representation purposes.

For each parcel, we also subtracted its minimum distance values between pairs of primary landmarks (auditory-somatomotor; visual-auditory; visual-somatomotor). By doing so, we obtained a representation of the position of each parcel along the axes formed by pairs of primary landmarks (Fig. 2*A*–*C*). We obtained values ranging from −182.2 to 182.2. A negative result represents that parcel *k* is located closer to the first primary landmark of the pair (distance to the first landmark < to the second), a positive value indicates that it is closer to the second landmark (distance to the first landmark > to the second), and a value close to 0 represents that it is approximately equally distant to both landmarks. This analysis provided equidistance values for each pair of sensory-motor landmarks.

We transformed these equidistance scores to absolute values, so that information about the direction of the imbalance (i.e., which landmark was closer) was removed. As a result, values further away from 0 represent greater imbalance between a pair of landmarks and closer to 0 represent balance. We normalized the values for each participant to a range of 0–1 using MinMaxScaler in scikit-learn, in order to make them comparable to global minimum distance values. The resulting normalized values represent parcel *k*'s equidistance between each pair of primary landmarks. We then averaged the three equidistances to obtain a general measure representing average equidistance of parcel *k* between any pair of primary landmarks (Fig. 1*C*).

#### Gradient and network analysis

We were interested in investigating how equidistance and minimum distance values vary across the cortex. To do that, we used the framework of the principal gradient of functional connectivity (Margulies et al., 2016), as represented in the map obtained from BrainSpace (Vos de Wael et al., 2020) in a previous investigation (Wang et al., 2023). This gradient was originally obtained by Margulies et al. by transforming high-dimensional matrices of connectivity between brain regions to low-dimensional components representing the similarity or dissimilarity in connectivity patterns between regions. The principal gradient of connectivity is a continuous variable that can be represented spatially in the brain (Fig. 3*A*), where the proximity of colors can be interpreted as greater similarity of connectivity patterns, and it captures the separation (i.e., dissimilarity in connectivity patterns) between unimodal sensory-motor systems and heteromodal regions. Additionally, we also explored the relationship between distance measures and myelination across the cortex (Text S1), which has been recently found to present a hierarchical pattern similar to the principal gradient of connectivity (Nishio et al., 2025).

We also investigated equidistance and global minimum distance values within the networks of a widely used parcellation based on intrinsic functional connectivity (Yeo et al., 2011). This parcellation provides a framework for examining large-scale cortical organization at multiple granularities and spatial scales: the 7-network solution captures broad functional systems, i.e., limbic, control, default, visual, somatomotor, and ventral and dorsal attention networks (Fig. S2*B*,*C*), while the 17-network solution offers finer subdivisions that distinguish subnetworks within these systems. We were particularly interested in variability within networks described as heteromodal (Margulies et al., 2016; Fig. S2*A*) and therefore focused our analyses on the subsystems of default mode and control networks described in the 17 network parcellation. We did not explore limbic subsystems due to their reduced number of parcels and low spatial variability.

For DMN, we focused on three subsystems: (1) core DMN comprising angular gyrus, medial prefrontal cortex, and posterior cingulate cortex; (2) medial temporal (MT) DMN focused on structures around the hippocampus and retrosplenial cortex, plus posterior parts of angular gyrus; and (3) frontotemporal (FT) DMN, including ventrolateral and dorsomedial prefrontal cortex, plus left lateral temporal cortex and ventral parts of angular gyrus (Fig. 3*A*). “Core DMN” is based on the nomenclature in Andrews-Hanna et al. (2010), and equivalent to Default-A in Kahali et al. (2021), based on the 17-network parcellation of Yeo et al. (2011). We use “MT DMN” for the medial temporal lobe subsystem in Andrews-Hanna et al. (2010) and Default-C in Kahali et al. (2021) and Yeo et al. (2011), while using “FT DMN” for the dorsal medial prefrontal cortex subsystem in Andrews-Hanna et al. (2010) and Default-B in Kahali et al. (2021) and Yeo et al. (2011).

For the control network, we also examined three subsystems: (1) Control-core focused on lateral prefrontal cortex, particularly inferior frontal sulcus and precentral gyrus, plus intraparietal sulcus; (2) Control-medial concentrated in posterior cingulate cortex and precuneus; and (3) Control-DMN encompassing frontal pole, dorsomedial prefrontal cortex, dorsal aspects of inferior parietal cortex, and posterior parts of inferior temporal cortex (Fig. 4*A*). These subsystems identified by the Yeo et al. (2011) parcellation allow us to test whether equidistance is linked to fine-grained functional specialization within a distributed network architecture. We use “Control-core” for the control subsystem referred to as FPCN-B in Dixon et al. (2018) and as Control-A in Kahali et al. (2021) and Yeo et al. (2011); “Control-medial” for the control subsystem referred to as Control-C in Kahali et al. (2021) and Yeo et al. (2011); and “Control-DMN” for the control subsystem referred to as FPCN-A in Dixon et al. (2018) and as Control-B in Kahali et al. (2021) and Yeo et al. (2011).

We adapted the original parcellation (400-parcel version) of the 7 and 17 networks (Schaefer et al., 2018) to the HCP multimodal parcellation map (Glasser et al., 2016), since global minimum distance and equidistance values had been obtained for this parcellation. We used a method based on the supplemental results of a previous investigation (Braver et al., 2021), available at http://osf.io/wv7ze/, inspired by a study using the Dice coefficient as a measure of spatial similarity between atlases defined in the same space (Lawrence et al., 2021). We quantified the correspondence between the original sets of parcels of each network and sets of HCP multimodal parcels using the Dice coefficient, choosing the set of parcels with the largest coefficient as the one with the best correspondence to be used in next steps of our analyses. Visual inspection confirmed that the sets of HCP multimodal parcels were equivalent to the original networks, and Dice values of the definitive sets of parcels selected ranged between 0.93 (for the right hemisphere of the visual network) and 0.646 (for the right hemisphere of Control-core).

#### Task activation

To explore the implications of equidistance on brain activation elicited by different types of tasks, we used the language task (containing story and math blocks; Binder et al., 2011) in the HCP dataset (Glasser et al., 2013), already analyzed using the HCP multimodal parcellation in a previous investigation (Wang et al., 2023).

The story blocks consisted of brief auditory stories, of different difficulties, including 5–9 sentences adapted from Aesop's fables, followed by a 2-alternative forced-choice question about the content. The math blocks included spoken arithmetic operations followed by a forced-choice decision between two possible results of the operation. Math and story blocks were similar in auditory input and attentional demands (Binder et al., 2011).

Data acquisition, preprocessing steps, and statistical analyses to extract activation levels for each parcel of the HCP multimodal parcellation were described previously (Barch et al., 2013; Wang et al., 2023). In brief, activation estimates for each participant were computed for the preprocessed functional time series from each run using a general linear model (GLM) implemented in FSL's FILM (FMRIB's Improved Linear Model with autocorrelation correction; Woolrich et al., 2001) for the surface. Story and math were the two predictors included in the model, covering the variable durations of the short story, question and response period, or an equivalent duration covering the math questions for the math predictor. Three contrasts were computed: story only, math only, and the linear contrast of story versus math. After model estimation, beta-weight images for the period of interest (story only and math only), contrasting the relevant time points with the fixation periods or implicit baseline, were used to capture the relevant pattern of activation of each task. Beta COPE maps were generated using custom-built MATLAB scripts after changing the data from CIFTI file format to the MATLAB workspace and correcting for the intensity bias field with an improved method (Glasser et al., 2016). Activation estimates on the cortical surface were averaged across vertices that shared the same parcel label in every subject. We used the sum of beta-values of activation for story and math tasks and the contrast of story > math in our analyses, following the approach used in a previous study (Wang et al., 2023).

### Statistical analysis

#### Relationship between global minimum distance and equidistances

To explore the differences between average equidistance and global minimum distance along the brain, we carried out two-sample *t* tests for each parcel of the whole brain across participants, controlling for multiple comparisons using Bonferroni’s correction and a *p* value threshold of 0.0001. We then calculated the effect sizes (Cohen's *d*) of these differences and used the resulting values for the visual representation in Figure 1*E*.

In order to explore the similarities between equidistances (equidistances between pairs of primary landmarks and average equidistance) and global minimum distance, we ran Pearson’s correlations between the measures (average values across participants) across all parcels of the brain, in both hemispheres separately. Results did not differ by hemisphere so we presented the mean correlation across hemispheres (Figs. 1*D*, 2*D*–*F*). We used a *p* value of 0.05 as threshold for significance, controlled for the spatial autocorrelation (SA) of the maps by generating 1,000 surrogates with the tools available in BrainSmash (Burt et al., 2020), and corrected for multiple comparisons using Bonferroni’s correction.

#### Global minimum distance and equidistance across the cortex

In order to explore the variation of distance values across the cortex, we carried out Pearson’s correlations between the distance measures (three equidistances between pairs of primary landmarks, average equidistance and global minimum distance) and the principal gradient of connectivity (Fig. 3, Fig. S1), using the procedure described above.

Finally, in order to account for the influence that global minimum distance and equidistance have on each-others relationship with the gradient, we calculated partial correlations between the gradient and equidistances (i.e., three equidistances between each pair of landmarks and average equidistance), controlling for global minimum distance, and between the gradient and global minimum distance, controlling for equidistance. We represented these partial correlations graphically by residualizing the gradient and equidistance for global minimum distance and the gradient and global minimum distance for equidistance. Again, results did not differ by hemisphere, so we used the mean correlation for visual representation (Fig. 3*B*,*C*; Fig. S1). Since partial correlations cannot be controlled for SA, we do not report nor interpret their *p* values of statistical significance.

We also compared equidistances (i.e., three equidistances between each pair of landmarks and average equidistance) and global minimum distance values between seven networks (Yeo et al., 2011), i.e., limbic, default, control, visual, somatomotor, ventral and dorsal attention networks. To do so, we applied mixed-effect models in R (https://www.R-project.org/) using the *lmer* function (from the lme4 package), with network as independent variable, distance value as dependent variable, and parcel number and participant as random effects (Fig. S2). Finally, we explored the influence of global minimum distance on the equidistance models by including it as an additional predictor (Fig. S2*D*), and the influence of average equidistance on the global minimum distance model by also including it as a predictor in that model.

#### Differences in distances between heteromodal subsystems

To explore the differences between heteromodal subsystems in average equidistance and minimum distance, we used mixed effects models. We included subsystems (core, MT and FT DMN, or Control-DMN, core and medial) as the independent variable, distance values (average equidistance or global minimum distance) as the dependent variable, and parcel number and participant as random effects (Fig. 4*B*,*C*). We also explored the influence of global minimum distance and average equidistance on each other's effects as described above.

We were also interested in investigating the degree of imbalance of DMN and control subsystems between any of the pairs of primary landmarks. To explore this, we built mixed effects models with value of equidistance between pairs of primary landmarks as the dependent variable, pairs of primary landmarks (auditory-somatomotor, visual-auditory, or visual-somatomotor) as the independent variable, and parcel number and participant as random effects (Figs. 4*D*, 5*D*). Additionally, we explored the influence of global minimum distance on the models by including it as an additional predictor.

The significance of all mixed models was assessed using type III analysis of variance with the Satterthwaite's method (*anova* function), with a *p* value of 0.05 as threshold of significance. If a model was significant, we carried out pairwise comparisons between levels of the independent variable in order to find out the source of the significant result. We used the *emmeans* function, applying the Kenward–Roger degrees-of-freedom method and a *p* value of 0.05 as threshold for significance adjusted using the Tukey method. We also investigated effect size of the statistically significant post hoc contrasts using Cohen's *d*, using a threshold of *d* > 0.8 indicating large and meaningful effects (Cohen, 1988).

#### Functional consequences of equidistance between pairs of primary landmarks

Finally, we explored the relationship between distance measures and brain activation elicited by language and math tasks. To do this, we carried out Pearson’s correlations between brain activation (i.e., sum of story and math tasks, and contrast of story > math) and global minimum distance and equidistances between pairs of landmarks across all participants. Analyses were restricted to parcels within an area comprising the default and control networks in the left hemisphere (Fig. 6). We did not explore average equidistance in this analysis because we found its effects to be highly dependent on global minimum distance (see Fig. 1*D* for correlation between global minimum distance and average equidistance and Fig. 3*B* for correlation between equidistance and principal gradient controlled by global minimum distance). Finally, we assessed statistical significance by using the FDR correction for the number of parcels and Bonferroni’s correction for the number of comparisons.

## Results

### Relationship between global minimum distance and equidistance

Our first analyses examined the relationship between global minimum distance to any sensory-motor landmark and the average degree of equidistance between pairs of these landmarks. We found significant positive correlations between global minimum distance and average equidistance in both hemispheres (LH: *r* = 0.778, *p* < 0.001; RH: *r* = 0.791, *p* < 0.001; Fig. 1*D*). Only equidistance between visual and auditory landmarks was significantly correlated with global minimum distance (LH: *r* = 0.665, *p* = 0.008; RH: *r* = 0.676, *p* = 0.008; Fig. 2*F*). Global minimum distance was not significantly correlated with equidistance between visual and somatomotor landmarks (LH: *r* = 0.521, *p* > 0.1; RH: *r* = 0.533, *p* > 0.1), nor with equidistance between auditory and somatomotor landmarks (LH: *r* = 0.286, *p* > 0.1; RH: *r* = 0.302, *p* > 0.1; Fig. 2*D*,*E*). All *p* values are corrected for SA using surrogate model-based nulls and for multiple comparison using Bonferroni correction. These results suggest that there are aspects of global functional brain organization that are not captured by global minimum distance.

**Figure 1.**
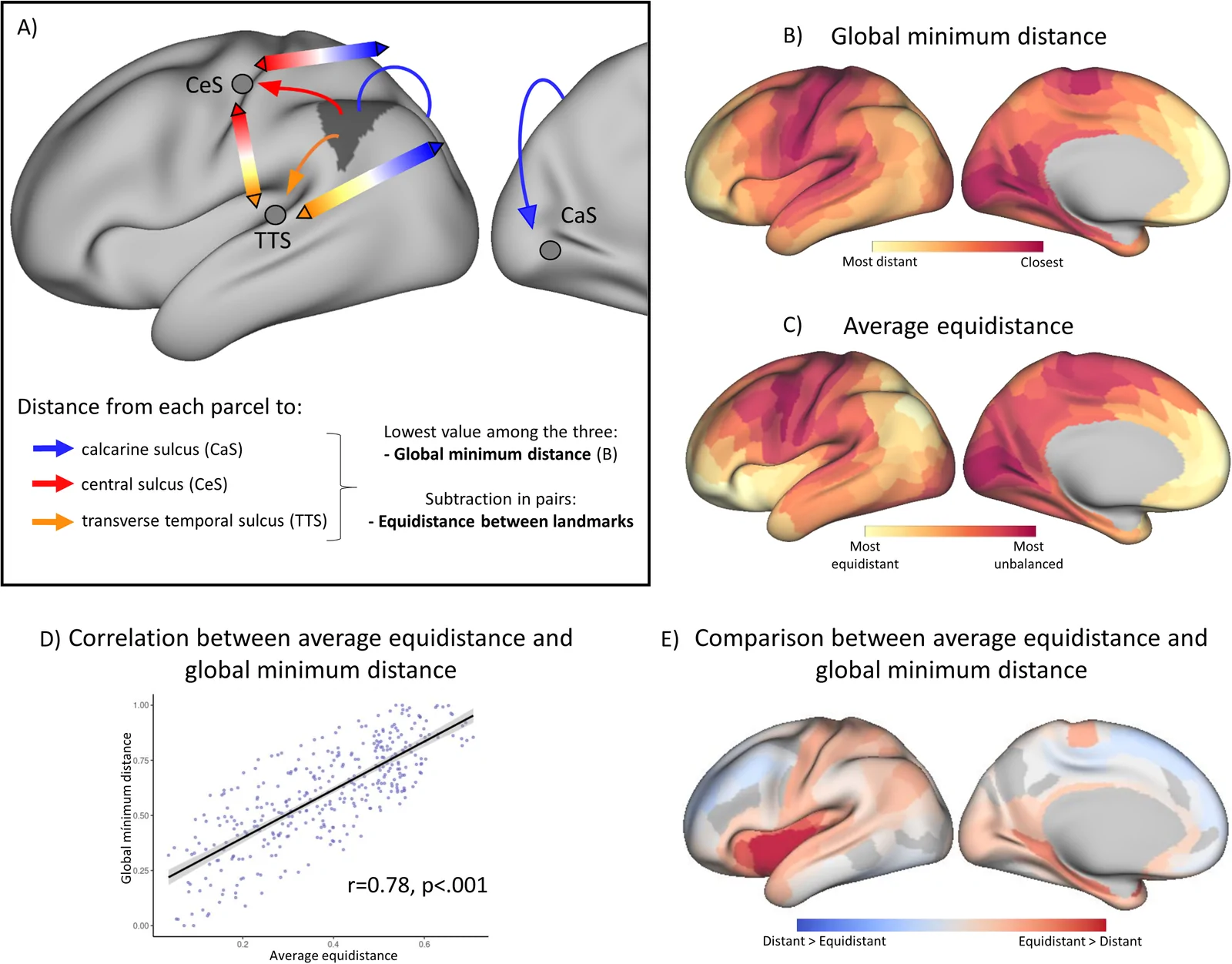
***A***, Distance from each parcel of the 360 in Glasser et al. (2016) to the three primary landmarks was extracted (CeS, central sulcus; TTS, temporal transverse sulcus; CaS, calcarine sulcus). The lowest value among the three was defined as the global minimum distance of each parcel, and values were normalized for each participant using a range of 0–1. The three distance values were subtracted in pairs to obtain equidistance between pairs of landmarks for each parcel. ***B***, Brain representation of global minimum distance. Bright yellow indicates parcels that are the most distant from primary landmarks (values closer to 0) and red indicates closer parcels (values closer to 1). ***C***, Brain representation of the average equidistance. This measure was obtained by transforming the three equidistances between specific pairs of primary landmarks to absolute values, averaging them and normalizing the result to a range of 0–1 for each participant. Bright yellow indicates parcels that are the most equidistant between primary landmarks (values closer to 0) and red indicates unbalanced parcels (values closer to 1). ***D***, Mean correlation between average equidistance and global minimum distance across two hemispheres, controlled for SA and multiple comparisons. Dots represent parcels. Results did not differ by hemisphere and thus they are represented in a single correlation plot. ***E***, Effect size (Cohen's *d*) of the comparison between average equidistance and global minimum distance for each parcel (two-sample *t* test). Blue colors indicate parcels that are more distant than equidistant, and red colors indicate parcels that are more equidistant than distant.

**Figure 2.**
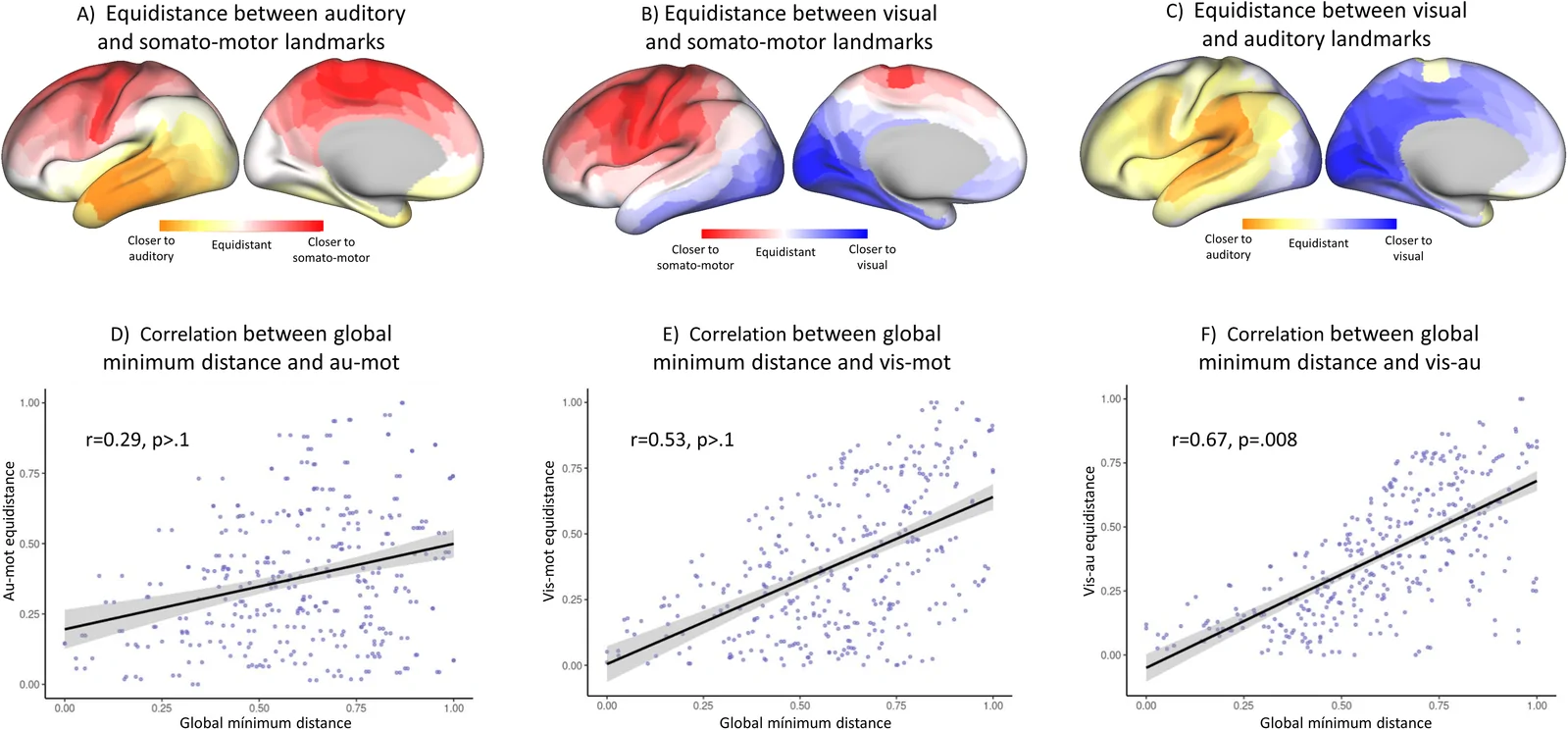
***A***, Brain representation of equidistance between auditory and somatomotor landmarks. Red indicates parcels closer to the somatomotor landmark, orange indicates parcels closer to the auditory landmark, and white indicates equidistant parcels. ***B***, Brain representation of equidistance between visual and somatomotor landmarks. Red indicates parcels closer to the somatomotor landmark, blue indicates parcels closer to the visual landmark, and white indicates equidistant parcels. ***C***, Brain representation of equidistance between auditory and visual landmarks. Orange indicates parcels closer to the auditory landmark, blue indicates parcels closer to the visual landmark, and white indicates equidistant parcels. Mean correlation across two hemispheres between global minimum distance and equidistance between the following primary systems: ***D***, auditory-somatomotor, ***E***, visual-somatomotor, and ***F***, visual-auditory. Dots represent parcels. Results are controlled for SA and for multiple comparisons. Results did not differ by hemisphere and thus they are represented in a single correlation plot. Au-mot, auditory-somatomotor; Vis-mot, visual-somatomotor; Vis-au, visual-auditory.

### Global minimum distance and equidistance across the cortex

Next, we examined the relationship between distance measures and the principal gradient of intrinsic connectivity, which explains the most variance in whole-brain resting-state fMRI. The principal gradient of intrinsic connectivity was significantly correlated with global minimum distance (LH: *r* = 0.509, *p* = 0.017; RH: *r* = 0.475, *p* = 0.019; Fig. 3*B*), but not equidistance (LH: *r* = −0.368, *p* = 0.089; RH: *r* = −0.345, *p* = 0.114; Fig. 3*C*). The correlation between the principal gradient and average equidistance fell to near zero when global minimum distance was controlled (LH: *r* = 0.052, RH: *r* = 0.058), but the correlation between the principal gradient and global minimum distance remained moderate even after controlling for equidistance (LH: *r* = −0.381; RH: *r* = −0.353; Fig. 2*C*). *p* values indicating significance are not reported for partial correlations because they cannot be corrected for spatial autocorrelation. Moreover, equidistances between specific pairs of primary landmarks were not significantly correlated with the principal gradient (auditory-somatomotor: *r* = 0.003, *p* > 0.1; visual-somatomotor: *r* = 0.38, *p* > 0.1; visual-auditory: *r* = 0.28, *p* > 0.1; Figure S1). All *p* values are corrected for SA using surrogate model-based nulls and for multiple comparison using Bonferroni’s correction. These analyses show that while the principal gradient of intrinsic connectivity, capturing the unimodal-to-heteromodal hierarchy, is linked to global minimum distance (as reported by Margulies et al., 2016), equidistance shows a different pattern.

**Figure 3.**
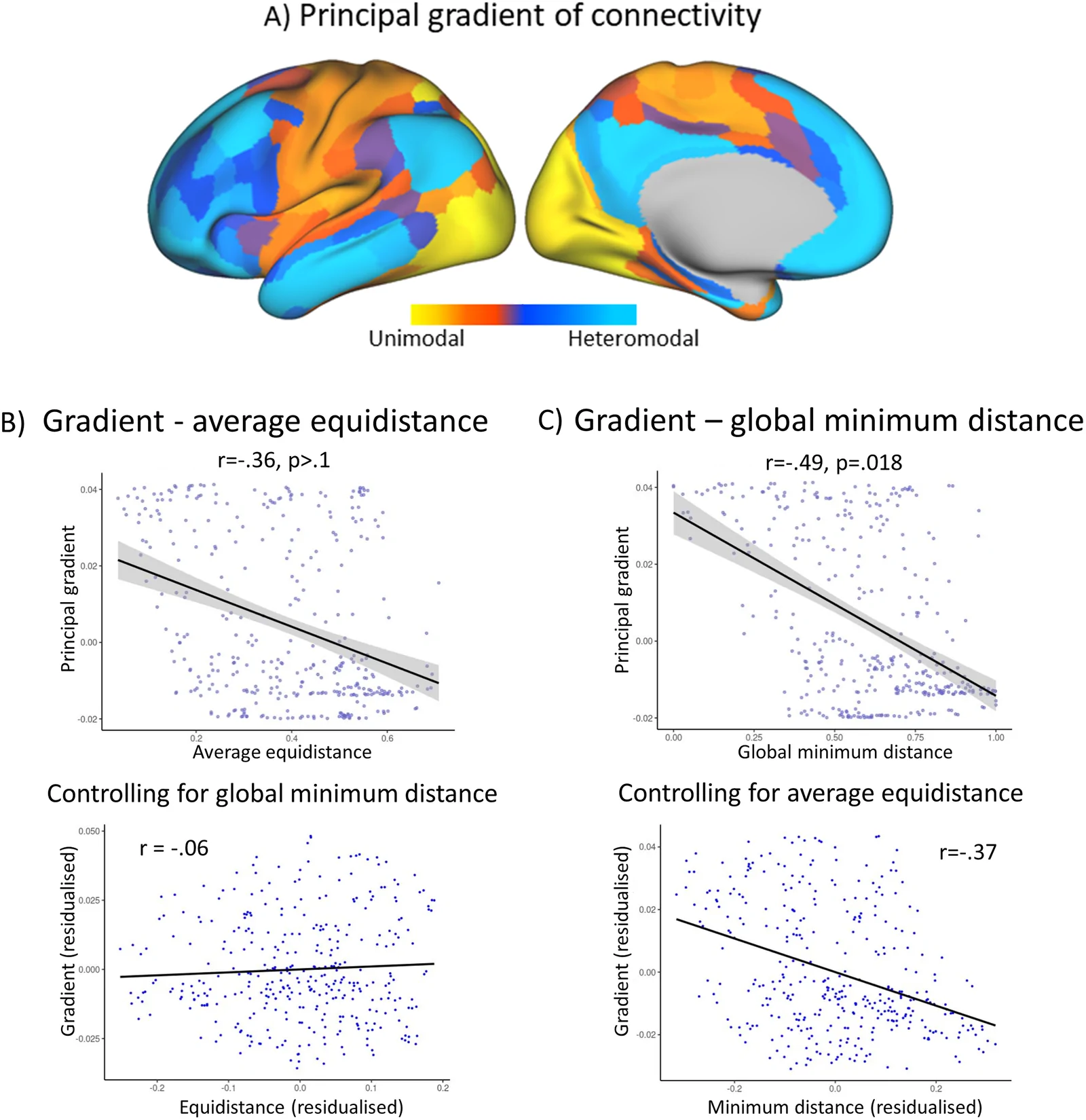
***A***, The principal gradient of intrinsic connectivity as described by Margulies et al. (2016). Yellow and orange colors indicate regions that are closer to the unimodal end of the gradient (lower gradient values), and blue indicates areas closer to the heteromodal end of the gradient (higher gradient values). ***B***, The left column shows correlations between the principal gradient of connectivity and average equidistance, corrected for SA and multiple comparisons. In the second row, correlations are controlled by global minimum distance. Dots represent parcels. Results did not differ by hemisphere and thus they are represented in a single correlation plot. ***C***, The right column shows correlations between the principal gradient of connectivity and global minimum distance to sensory-motor landmarks, corrected for SA and multiple comparisons. In the second row, correlations are controlled by equidistance. Dots represent parcels. Results did not differ by hemisphere and thus they are represented in a single correlation plot.

### Network differences in global minimum distance and average equidistance

We explored differences in equidistance and global minimum distance between seven canonical resting-state networks: limbic, control, DMN, ventral and dorsal attention, and visual and somatomotor networks (Yeo et al., 2011). DMN, limbic, and control networks were significantly more distant from primary landmarks than the rest of networks (*F*_(6,326.03)_ = 35.76, *p* < 0.0001; see Table S1 for pairwise statistics). These differences remained significant even after controlling for equidistance by including it as a predictor in the model. For average equidistance, somatomotor and visual networks were significantly more unbalanced than other networks. Ventral attention network was more balanced in its proximity to primary landmarks than the somatomotor network, but more unbalanced than the limbic and control networks (*F*_(6,326.01)_ = 35.78, *p* < 0.0001; see Table S1 for pairwise statistics). However, when controlling for global minimum distance by including it as a predictor in the model, only the differences between visual-dorsal attention and visual-limbic networks survived (Fig. S3). This shows that heteromodal networks, including DMN, control and limbic, are relatively distant and equidistant from primary landmarks, yet many of the network differences seen in average equidistance reflect covariation with global minimum distance.

Heteromodal networks were not consistently equidistant between specific pairs of primary landmarks. For instance, DMN was more equidistant than the frontoparietal control network in the visual-somatomotor axis, but more unbalanced than the limbic network in the visual-auditory axis, and more unbalanced than the visual network between auditory and somatomotor primary landmarks (Fig. S3).

### Differences in distances between DMN subsystems

Next, we explored whether distance to sensory landmarks varied across the subsystems of heteromodal networks [as defined by Yeo et al. (2011) 17-network parcellation of resting-state fMRI data]. We investigated differences between DMN and control network subsystems in global minimum distance, average equidistance, and equidistance between pairs of landmarks in order to understand whether topographical differences might be contributing to previously described functional variation within these networks.

We found no significant differences between the three DMN subsystems in average equidistance (*F*_(2,40.041)_ = 0.85, *p* = 0.435; Fig. 4*B*), but they significantly differed in global minimum distance (*F*_(2,74.968)_ = 9.10, *p* = 0.0002; Fig. 4*C*). Specifically, MT DMN was significantly closer to primary systems than core and FT DMN (core vs MT: estimate = −0.303, *z* = −4.227, *p* = 0.0001; FT vs MT: estimate = −0.255, *z* = −3.326, *p* = 0.002; Tukey-adjusted and Cohen's *d* > 0.8). These results were still significant when controlling for equidistance.

**Figure 4.**
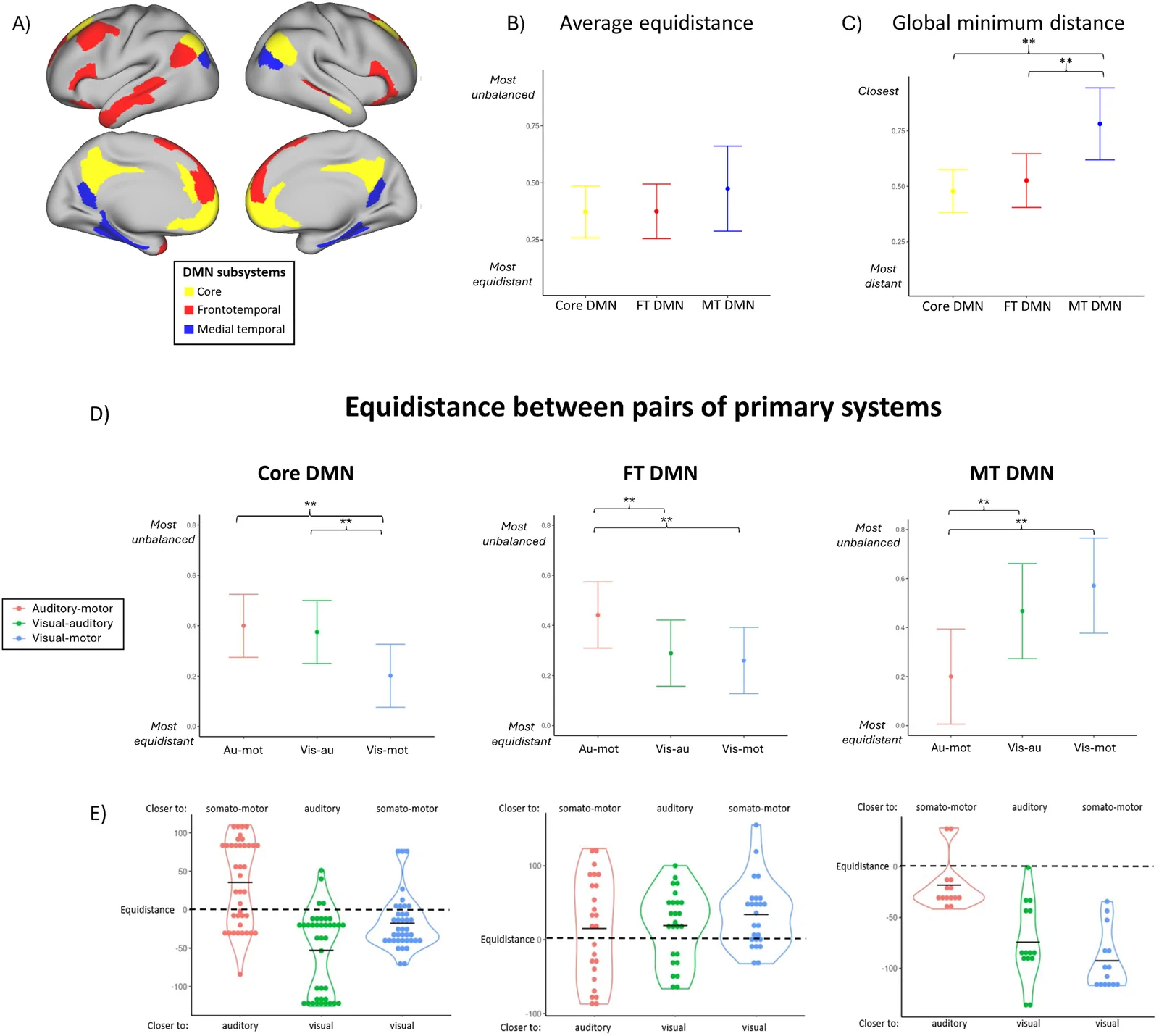
***A***, Location of default mode network (DMN) subsystems as described in a 17 network subdivision (Yeo et al., 2011) in 360 brain parcels (Glasser et al., 2016). Core DMN in yellow, frontotemporal (FT) DMN in red, and medial temporal (MT) DMN in blue. Estimated marginal means and 95% confidence intervals obtained from linear mixed models using subsystems (core, FT, and MT DMN) or primary axes (auditory-somatomotor, visual-auditory, visual-somatomotor) as independent variable, and values of the following metrics in all participants and 360 brain parcels (Glasser et al., 2016) as dependent variables: ***B***, average equidistance, ***C***, global minimum distance, and ***D***, equidistance between pairs of primary landmarks. Bars and asterisks indicate significant pairwise differences between subsystems or axes of pairs of primary landmarks (*p* < 0.05, Tukey-adjusted, Cohen's *d* > 0.8). ***E***, Observed values of distance between pairs of primary landmarks in parcels within subsystems, indicating direction of imbalance. Each dot represents a parcel. The short horizontal lines indicate the mean value within each axis of pairs of primary landmarks, and the dotted lines represent the equidistance point. Au-mot, auditory-somatomotor; Vis-au, visual-auditory; Vis-mot, visual-somatomotor.

We also investigated differences between DMN subsystems in the equidistance between pairs of primary systems. Core DMN showed significant differences in equidistance when this metric was computed for different pairs of primary systems (*F*_(2,92.956)_ = 13.77, *p* < 0.0001): it was more equidistant for visual and somatomotor cortex than for other pairs of primary landmarks (auditory-somatomotor vs visual-somatomotor: estimate = 0.222, *z* = 170.598, *p* < 0.0001, visual-auditory vs visual-somatomotor: estimate = 0.199, *z* = 153.250, *p* < 0.0001; Tukey-adjusted and Cohen's *d* > 0.8; Fig. 4*D*). FT DMN also showed significant differences between different pairs of primary systems (*F*_(2,54.385)_ = 7.82, *p* = 0.001): it showed greater imbalance in the auditory-somatomotor axis than for the other pairs (auditory-somatomotor vs visual-auditory: estimate = 0.167, *z* = 116.525, *p* < 0.0001; auditory-somatomotor vs visual-somatomotor: estimate = 0.209, *z* = 145.177, *p* < 0.0001; Tukey-adjusted and Cohen's *d* > 0.8; Fig. 4*D*), reflecting proximity of the parcels within this subsystem to the auditory or somatomotor cortex (Fig. 4*E*). Finally, MT DMN also showed significant differences between pairs of primary systems (*F*_(2,31.822)_ = 30.93, *p* < 0.0001): it was significantly more equidistant to auditory-motor inputs than for comparisons including the visual landmark (auditory-somatomotor-visual-auditory: estimate = −0.313, *z* = −198.526, *p* < 0.0001; auditory-somatomotor-visual-somatomotor: estimate = −0.408, *z* = −258.479, *p* < 0.0001; Tukey-adjusted and Cohen's *d* > 0.8; Fig. 4*D*), reflecting the proximity of its parcels toward the visual cortex (Fig. 4*E*). All results remained significant when controlling for global minimum distance by adding this variable as a predictor in the model. Therefore, DMN subsystems have a distinctive pattern of distance with primary systems: core DMN is located in cortical positions equidistant between somatomotor and visual cortex; FT DMN is composed of parcels that are very unbalanced toward the auditory or somatomotor primary systems, and MT DMN is closer to visual cortex as a whole.

### Differences in distance between control network subsystems

We employed the same approach to examine whether subsystems of the control network occur at different distances to sensory landmarks. We found significant differences between the three control subsystems both in equidistance (*F*_(2,25.991)_ = 4.51, *p* = 0.02; Fig. 5*B*) and in global minimum distance (*F*_(2,26.01)_ = 8.41, *p* = 0.002; Fig. 5*C*). Control-DMN was significantly more equidistant overall than control-medial (estimate = −0.262, *z* = −2.529, *p* = 0.031; Fig. 5*B*), although this result was not significant if global minimum distance was included in the model as a predictor. Moreover, Control-DMN showed higher global minimum distance to sensory-motor landmarks than control-core and control-medial subsystems (Control-core vs Control-DMN: estimate = 0.247, *z* = 4.608, *p* < 0.0001; Control-DMN vs Control-medial: estimate = −0.364, *z* = −4.831, *p* < 0.0001; Tukey-adjusted and Cohen's *d* > 0.8; Fig. 5*C*), and these results remained significant when controlling for equidistance.

**Figure 5.**
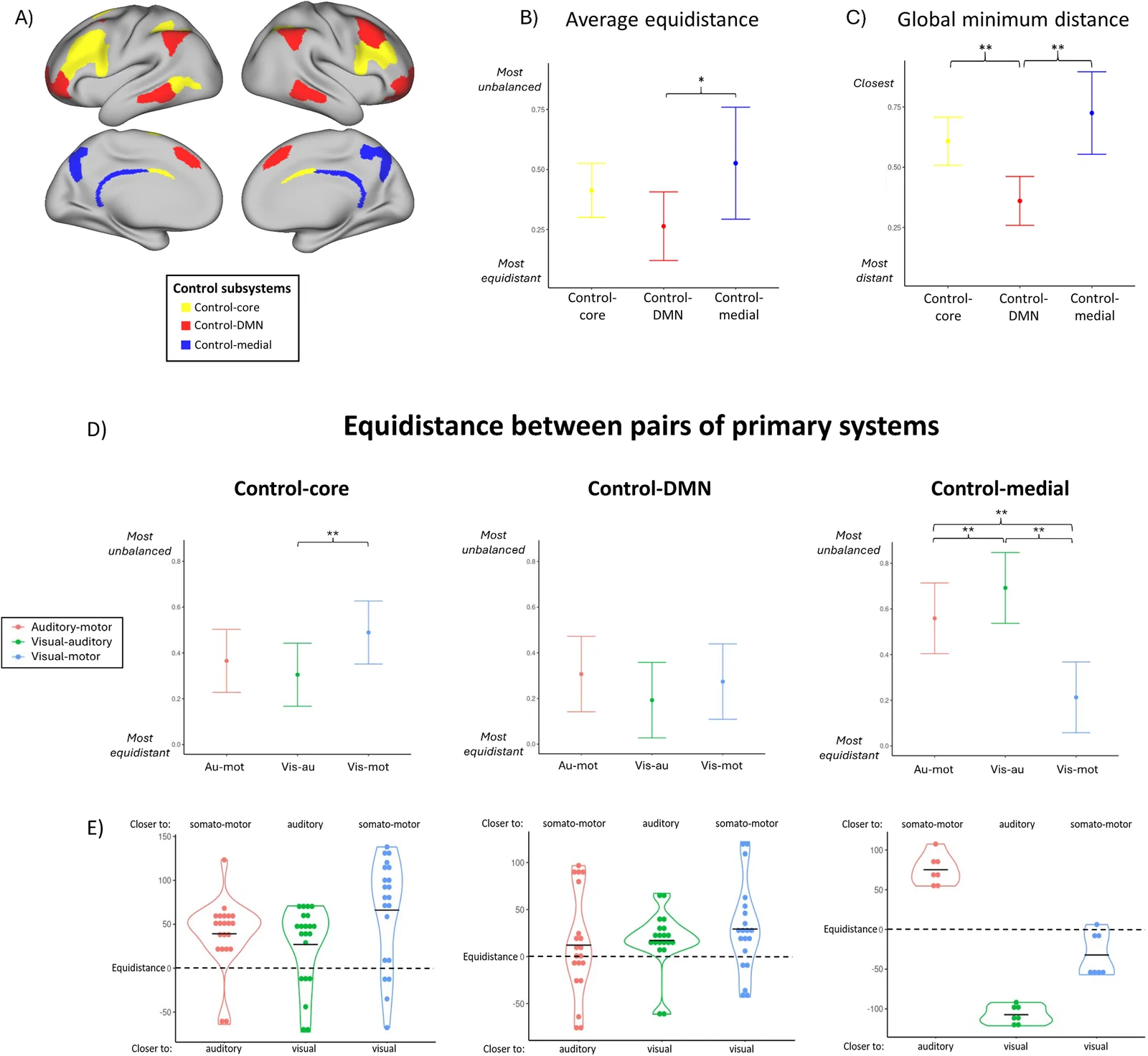
***A***, Location of control subsystems as described in a 17 network subdivision (Yeo et al., 2011) in 360 brain parcels (Glasser et al., 2016). Control-core in yellow, Control-DMN in red, and Control-medial in blue. Estimated marginal means and 95% confidence intervals obtained from linear mixed models using subsystems (Control-DMN, core, and medial) or primary axes (auditory-somatomotor, visual-auditory, visual-somatomotor) as independent variable, and values of the following metrics in all participants and 360 brain parcels (Glasser et al., 2016) as dependent variables: ***B***, average equidistance, ***C***, global minimum distance, and ***D***, equidistance between pairs of primary landmarks. Bars and asterisks indicate significant pairwise differences between subsystems or axes of pairs of primary landmarks (*p* < 0.05, Tukey-adjusted, Cohen's *d* > 0.8). ***E***, Observed values of distance between pairs of primary landmarks in parcels within subsystems, indicating direction of imbalance. Each dot represents a parcel. The short horizontal lines indicate the mean value within each axis of pairs of primary landmarks, and the dotted lines represent the equidistance point. Au-mot, auditory-somatomotor; Vis-au, visual-auditory; Vis-mot, visual-somatomotor.

We also investigated differences in equidistance between control subsystems across pairs of primary systems. Control-core showed significant differences in equidistance to different pairs of primary regions (*F*_(2,48.72) _= 8.97, *p* = 0.0005): it was significantly more equidistant on the visual-auditory axis than the visual-somatomotor axis (estimate = −0.189, *z* = −134.013, *p* < 0.0001, Tukey-adjusted and Cohen's *d* > 0.8; Fig. 5*D*), reflecting its relative proximity to somatomotor regions. Control-medial also showed significant differences between pairs of primary regions (*F*_(2,18)_ = 24.32, *p* < 0.0001), being most balanced on the visual-somatomotor axis, least balanced on the visual-auditory axis, and intermediate on the auditory-somatomotor axis estimates ranging from –0.17 to 0.55 and *z*-values from –75 to 246 (all Tukey-adjusted *p* < 0.0001, Cohen's *d* > 0.8; Fig. 5*D*). This pattern reflects the control-medial subsystem's relative proximity to visual cortex and distance from auditory cortex. All results remained significant when controlling for global minimum distance by adding this variable as a predictor in the models. The Control-DMN subsystem was equally equidistant for all pairs of landmarks (*F*_(2,45.889)_ = 2.83, *p* = 0.07), suggesting it is uniquely positioned between all primary systems (Fig. 5*D*,*E*).

### Functional consequences of equidistance between pairs of primary landmarks

Finally, to understand the potential functional consequences of geodesic distance, we explored the relationship between individual differences in brain activation elicited by language and math tasks and their contrast with variation in global minimum distance or equidistance. We restricted our equidistance analyses to the three equidistances between pairs of primary landmarks because the effects of average equidistance were highly dependent on global minimum distance (see Fig. 1*D* for correlation between global minimum distance and average equidistance and Fig. 3*B* for correlation between equidistance and principal gradient controlled by global minimum distance).

**Figure 6.**
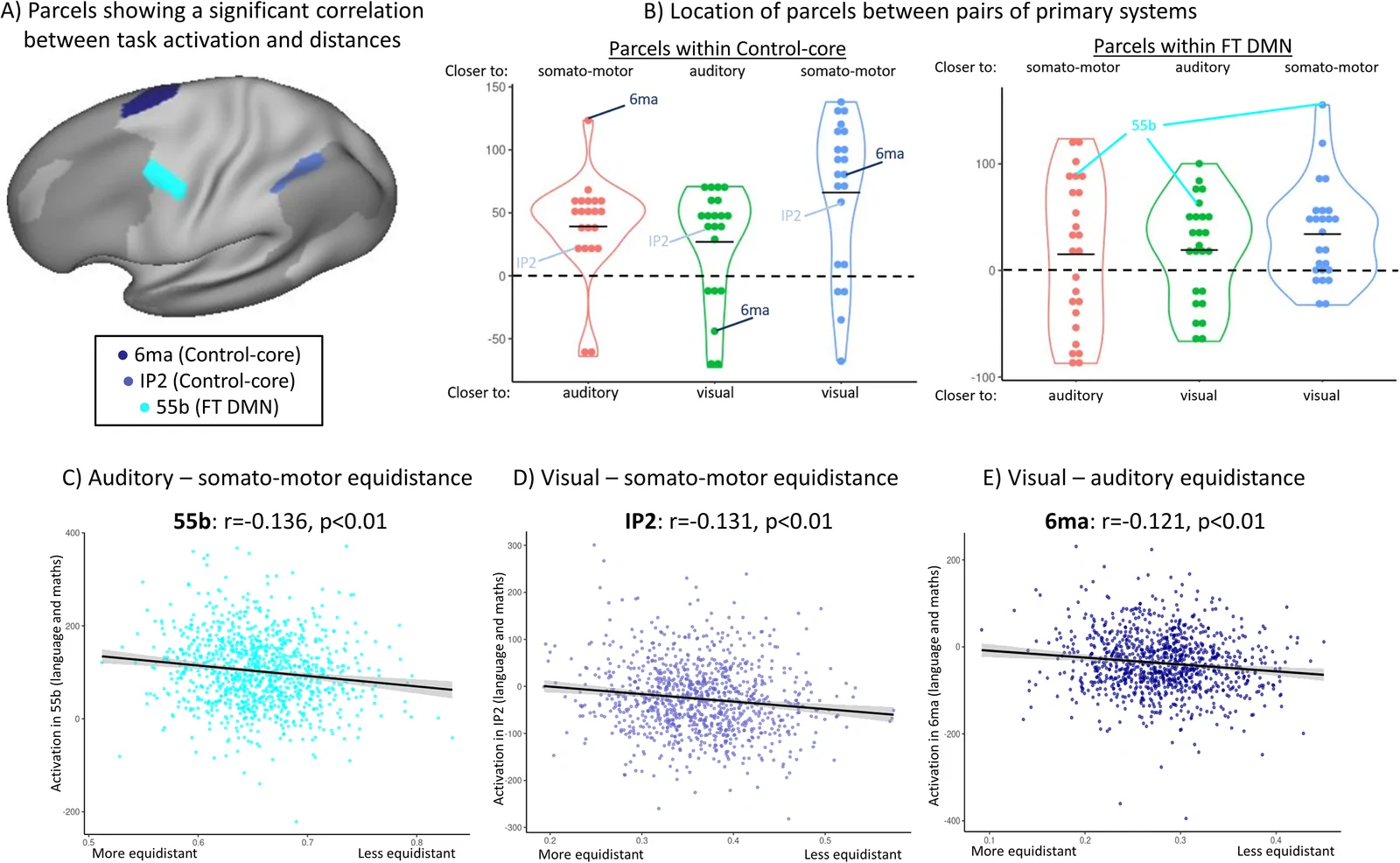
***A***, Parcels within default and control networks in the left hemisphere showing a significant correlation between task activation (sum of story and math vs rest) and the three equidistances between pairs of primary landmarks, corrected for number of parcels and comparisons. Networks of interest (DMN and control) are represented in gray, a premotor region (55b) is cyan, anterior inferior parietal sulcus (IP2) is light blue, and a paracentral/cingulate region (6ma) is dark blue. We use the nomenclature found in the original parcellation (Glasser et al., 2016). ***B***, Location of parcels in relation to the three equidistances between pairs of primary landmarks, within their corresponding subsystem (Control-core and FT DMN). ***C***, Significant correlation between auditory-somatomotor equidistance and activation in 55b (cyan). ***D***, Significant correlation between visual-somatomotor equidistance and activation in IP2 (light blue). ***E***, Significant correlation between visual-auditory equidistance and activation in 6 ma (dark blue).

We extracted correlations between the three equidistances and brain activation elicited by language and math tasks for the parcels of heteromodal networks (DMN and control), corrected for multiple comparisons. Parcel 55b within FT DMN (Fig. 6*A*,*B*) showed a significant correlation between activation and equidistance in the auditory-somatomotor axis (*r* = −0.136, *p* < 0.01, FDR and Bonferroni corrected; Fig. 6*C*). Individuals who showed more equidistance for this parcel also showed stronger activation in response to auditory input in these tasks. This parcel was relatively close to the landmark for primary somatomotor cortex; it showed more activation when it was more equidistant with auditory inputs, consistent with FT DMN's function in sensory integration.

There were also two significant correlations with parcels in the core subsystem of the control network. Parcel IP2 (Fig. 6*A*,*B*) showed a significant correlation between activation and equidistance on the visual-somatomotor axis (*r* = −0.131, *p* < 0.01, FDR and Bonferroni corrected; Fig. 6*D*). Finally, parcel 6ma (Fig. 6*A*,*B*) showed a significant correlation between activation and equidistance in the visual - auditory axis (*r* = −0.121, *p* < 0.01, FDR and Bonferroni corrected; Fig. 6*E*). These parcels also showed more activation with greater equidistance between primary landmarks, consistent with the core control network's transmodal function.

Notably, all three significant parcels were located at the edges of heteromodal networks, rather than in their deep cores (Fig. 6*A*). This topographical position means they can fall at the extremes of equidistance, being relatively close to one primary system while more distant from the others (Fig. 6*B*). Our results suggest that when such modality-biased parcels are more evenly balanced between inputs, they show stronger task-related activation. In other words, equidistance appears to enhance the responsiveness of heteromodal border zones, supporting the idea that spatial embedding shapes the capacity of DMN and control systems to integrate across modalities.

We explored the relationship between equidistance and brain activation elicited by maths or story tasks alone, and the results obtained using the sum of the two tasks were largely replicated (Fig. S3). Correlations between brain activation and global minimum distance were also explored within the same DMN and control network regions, but no significant results were found. A previous study (Wang et al., 2023) did find opposing relationships between geodesic distance and the BOLD response in auditory cortex (smaller distance linked to more activation) and the language network (greater distance linked to more activation). We also explored the relationship between equidistance and the difference in brain activation elicited by story and math (story > math contrast). Three parcels showed significant correlations between the contrast values and two of the three equidistances, suggesting consistency across landmarks of interest. However, they were not clearly linked to associations of equidistance with either task independently (see Fig. S3 for details). Finally, in order to check how the normalization procedure influenced the outcome of the analysis, we explored correlations between brain activation and raw (not normalized) values of equidistance. These analyses replicated the results we obtained using normalized equidistances: parcels 6ma, IP2, and 55b showed significant negative correlations with task activation.

## Discussion

A fundamental question in systems and cognitive neuroscience is how the large-scale geometry of the cortex shapes functional organization. Sensory and motor cortices provide fixed spatial anchors, yet association regions must flexibly integrate information from multiple modalities while supporting diverse cognitive operations. Heteromodal networks, such as the DMN and frontoparietal control network, are distant from sensory-motor cortices, reflecting their integrative role. However, less is known about how their relative position with respect to multiple sensory-motor landmarks contributes to functional specialization. This study addressed this question by quantifying the equidistance of each cortical parcel to pairs of primary sensory-motor landmarks, asking how this topographic balance relates to both network structure and cognitive function.

We show that heteromodal networks, including the default mode and frontoparietal control system, occupy cortical positions that are not only distant from primary sensory-motor regions but also relatively balanced between them. This supports the notion that these networks are spatially poised for integrative processing, sitting at the intersection of multiple unimodal systems. While average equidistance is tightly linked to global minimum distance, finer-grained analyses show that subnetworks within DMN and control systems exhibit distinct equidistance profiles. Functional heterogeneity within these networks might therefore arise, at least in part, from their relative positioning with respect to different sensory-motor landmarks. Critically, parcels with higher equidistance were more strongly engaged during language comprehension and math tasks, indicating that being spatially balanced across inputs enhances the responsiveness of heteromodal regions. Together, these findings establish equidistance as a complementary dimension of cortical organization, shaping not only network placement but also functional engagement.

We found that average equidistance and minimum distance are highly correlated, and the relationship between average equidistance and the principal gradient of connectivity is attenuated when the influence of global minimum distance is controlled. However, the three equidistances between pairs of primary landmarks are not significantly related to global minimum distance nor to the principal gradient of connectivity. Moreover, the differences in equidistances between networks (Yeo et al., 2011) remain significant after controlling for global minimum distance, as opposed to the reductions seen in average equidistance. Therefore, while average equidistance largely tracks global minimum distance, pairwise equidistance offers a finer-grained view of heteromodal network organization. It offers nuanced meaning, representing not only whether a region is balanced, but which primary anchors it is balanced between, or biased toward. Subnetworks within the DMN and control systems occupy distinct spatial positions relative to visual, auditory, and somatomotor regions, and these geometric relationships are likely to shape their functional specialization. Brain areas are strongly connected to nearby cortical regions through short-range *U*-shaped fibers (Catani and Thiebaut de Schotten, 2012; Ercsey-Ravasz et al., 2013) while also maintaining long-range links with distant nodes of the same functional network (Sepulcre et al., 2012). This combination of local and global connectivity implies that pairwise equidistance supports flexible integration across modalities: regions can preferentially draw on inputs from the modalities they lie nearest to, while long-range connections coordinate activity across subnetworks to form coherent large-scale systems. In this way, fine-grained spatial embedding provides a structural basis for the balance between specialization and integration that characterizes heteromodal cortex.

Within the DMN, spatial variation between parcels establishes functional distinctions. The core subsystem shows equidistance between motor and visual landmarks, supporting internally directed cognition and cross-modal integration (Andrews-Hanna, 2011; Smallwood et al., 2021; Zhang et al., 2022). The medial temporal (MT) subsystem lies nearer to the visual cortex, consistent with scene construction and spatial navigation (Hassabis and Maguire, 2007; Ranganath and Ritchey, 2012; Gonzalez Alam et al., 2024). The frontotemporal (FT) subsystem, associated with semantic and social cognition (Andrews-Hanna et al., 2010; Davey et al., 2016), is positioned close to auditory or somatomotor regions, reflecting modality-specific contributions to its functional repertoire (Shao et al., 2024). By recruiting DMN parcels near auditory and motor regions for language and temporal context processing, semantic and social functions can be segregated from other DMN operations, such as memory retrieval during mind-wandering, supporting task-focused cognition (Zhang et al., 2022). At the same time, these distinct parcels remain interconnected, enabling global integration and highlighting a fine-grained topography of specialization within the DMN.

A similar diversity exists in the control network. The core control subsystem is closer to motor regions, fitting with its role in controlling actions and eye movements (Dixon et al., 2018). The control-DMN subsystem is more distant from unimodal landmarks and relatively equidistant overall, aligning with its proposed role in regulating heteromodal networks such as the DMN (Dixon et al., 2018; Gonzalez Alam et al., 2022). The control-medial subsystem is closer to visual regions and farther from somatomotor areas, consistent with its role in controlling visuospatial processing (Kravitz et al., 2011). This spatial segregation supports flexible cognitive control: tasks that rely primarily on abstract knowledge, such as recognizing objects, understanding words, or reasoning about social interactions, benefit from segregating semantic information from spatial context. Conversely, tasks like navigation or object search require integration of semantic and spatial information. Consequently, while a “core” multiple-demand network has been shown to activate in response to cognitive demands across tasks, the pattern of control recruitment can also vary as different subnetworks are engaged depending on the specific demands (Assem et al., 2020). These principles provide a mechanistic account of partial functional overlap between cognitive domains and networks, demonstrating how subnetwork-specific spatial embedding shapes the recruitment and integration of unimodal inputs.

The spatial embedding of heteromodal networks also has direct functional consequences. We found that individual differences in equidistance between pairs of primary landmarks predicted activation strength in both DMN and control parcels during story comprehension and math tasks. Parcels located at the edges of these networks, closer to a specific input modality, showed stronger activation when they were more equidistant from relevant inputs, suggesting that balanced access across modalities enhances engagement. This pattern highlights how equidistance supports integrative processing: parcels positioned to integrate multiple input streams can flexibly contribute to diverse cognitive tasks. Consistent with prior work (Wang et al., 2021), stronger integration across sensory modalities is associated with enhanced general cognitive ability, whereas greater segregation reflected in parcels biased toward a single input may support crystallized intelligence and processing speed. These findings provide a principled link between cortical geometry, local integration and segregation, and individual differences in cognitive function, illustrating how the fine-grained topography of heteromodal networks shapes task-specific recruitment. This could have implications for aging and disease—the brain changes produced by these processes (Pini et al., 2016) could alter the cortical configuration of distances and have relevant implications for cognitive function.

Several limitations of the study should be acknowledged. First, we assessed functional relevance in only a limited set of tasks. While parcels with higher equidistance showed greater activation during language comprehension and mathematical reasoning, different patterns might emerge in lower-level perceptual tasks, where closer proximity to unimodal regions may be advantageous. This raises the possibility that equidistance carries both costs and benefits, depending on the cognitive demands placed on a region. Second, it might be interesting to examine how equidistance–function relationships vary across the cortical surface, since in temporal and parietal regions, individual differences in equidistance can be interpreted as the extent to which regions fall between different sensory-motor systems, while in pre-rolandic cortex, this is no longer true—instead, equidistance is highly influenced by distance from motor and/or auditory cortex. Moreover, other cortical regions such as those comprising the limbic network may be poorly captured by their equidistance between the three primary landmarks selected in our study alone, due to their proximity to primary olfactory regions. Thus, it might be helpful to model the olfactory system as another primary landmark. Finally, our functional analyses were based on BOLD activation, which can have multiple interpretations, rather than direct measures of task performance. This limits our ability to determine whether equidistance relates to behavioral outcomes and specific cognitive functions. Future work incorporating a broader range of task domains and linking spatial metrics more directly to performance will be essential for clarifying the adaptive and potentially maladaptive consequences of equidistant positioning in cortical networks.

In conclusion, our findings show that the cortical organization of heteromodal networks is shaped by both distance from and relative balance between primary sensory-motor landmarks. Subnetwork-specific spatial embedding reflects functional specialization within networks and underpins the integration and segregation of information across tasks. Pairwise equidistance predicts task-evoked activation, linking cortical geometry directly to cognitive function, and highlighting a fine-grained mechanism by which the brain orchestrates flexible, context-dependent recruitment of distributed heteromodal systems.
